# Effects of resveratrol and oxyresveratrol on hippocampal cell death induced by kainic acid

**DOI:** 10.1080/19768354.2019.1620853

**Published:** 2019-05-31

**Authors:** Hee-Jung Lee, Jing-Hui Feng, Su-Min Sim, Soon-Sung Lim, Jae-Yong Lee, Hong-Won Suh

**Affiliations:** a Department of Pharmacology, Institute of Natural Medicine, College of Medicine Hallym University, Chuncheon, Republic of Korea; b Department of Food Science and Nutrition, Hallym University, Chuncheon, Republic of Korea; c Department of Biochemistry, College of Medicine, Hallym University, Chuncheon, Republic of Korea

**Keywords:** Oxyresveratrol, Kainic acid, cell death, hippocampus, FoxO3a

## Abstract

In the present study, we have examined the possible neuroprotective effects of resveratrol and oxyresveratrol against kainic-acid (KA)-induced hippocampal neuronal cell death. Either resveratrol or oxyresvertrol was orally administered 30 min prior to intracerebroventricular (i.c.v.) administration with KA (0.05 μg). Oral pretreatment with oxyresveratrol (50 mg/kg) significantly protected KA-induced hippocampal CA3 neuronal cell death. However, the same dose (50 mg/kg) or a higher dose (100 mg/kg) pretreatment with resveratrol did not affect KA-induced hippocampal neuronal cell death. Furthermore, the i.c.v. pretreatment with 30 μg of oxyresveratrol or resveratrol did not show the protective effect against KA-induced hippocampal neuronal cell death. In the immunohistochemical analysis, FoxO3a and pFoxO3a expressions in the hippocampal CA3 region were significantly increased 30 min after KA administration. Oral pretreatment with oxyresveratrol (50 mg/kg) significantly reduced KA-induced Forkhead homeobox type O3a (FoxO3a) and pFoxO3a expression in CA3 region of the hippocampus, suggesting that oxyresveratrol may exert a neuroprotective effect against KA-induced hippocampal neuronal cell death by reducing the levels of FoxO3a and pFoxO3a protein expression in the hippocampal CA3 region. Furthermore, it is suggested that the neuroprotective effect of orally administered oxyresveratrol against KA-induced neurotoxicity might be possibly mediated by some metabolites rather than direct action of oxyresveratrol on the central nervous system.

## Introduction

Kainic acid (KA) is a non-degradable analog of glutamate and is 30-fold more potent in neurotoxicity than glutamate. This neuroexcitant can bind to the AMPA/KA receptors, which are subtypes of the ionotropic glutamate receptors in the brain (Bleakman and Lodge 1998). Activation of KA receptor has been shown to elicit a number of cellular events, including the increase in intracellular Ca²^+^, production of reactive oxygen species, and other biochemical events leading to neuronal cell death (Sun et al. 1992; Cheng and Sun 1994; Gluck et al. 2000; Candelario-Jalil et al. 2001; Milatovic et al. 2002). Neuronal cell death occurred by KA elicits severe status epilepeticus in the pyramidal layer of the hippocampal CA3 region when KA is administered i.c.v (Sperk 1994). It may be because of excessive activation of neurons by excitatory neurotransmitters (e.g. glutamate), which are massively released as a consequence of energy depletion and which result in excitotoxic neuron death (Beal 1992).

Resveratrol (5-[(E)-2-(4-hydroxyphenyl)-ehenyl] benzene-1,3-diol) is a phytophenol present in high amounts in red grapes and wine. It has been suggested that resveratrol is largely responsible for the cardiovascular health benefits of red wine (Bradamante et al. 2004). Although there is as yet no direct evidence that dietary supplementation with resveratrol is effective in protecting against or treating neurological disease, an increasing number of studies in animal models have demonstrated neuroprotective effects of resveratrol. Administration of resveratrol to rats reduces ischemic damage to the brain in a model of stroke (Huang et al. 2001) and also protects spinal cord neurons against ischemic injury (Kaplan et al. 2005). Resveratrol can protect cultured neurons against nitric oxide-mediated oxidative stress-induced death (Bastianetto et al. 2000). In addition to reseveratrol, oxyresveratrol, hydroxystilbenes naturally occurring polyphenolic compounds, are well-known for their free radical scavenging properties (Lorenz et al. 2003; Huang et al. 2010). Recently it was reported that oxyresveratrol is a potent antioxidant (Lorenz et al. 2003). Oxyresveratrol is readily available from mulberry wood (*Morus alba* L.). Despite its better solubility in aqueous solutions and less cytotoxicity, yet it is pharmacologically less investigated. Moreover, research has shown that oxyresveratrol is transported into tissues at high rates resulting in a bioavailability of about 50% (Qiu et al. 1996). Such properties and its high solubility in aqueous solutions and low general toxicity makes oxyresveratrol potentially superior to resveratrol for neuroprotective studies. However, the exact roles of resveratrol and oxyresveratrol against KA-induced hippocampal neuronal toxicity have not been characterized yet. Thus, the present study was designed to examine the effects of resveratrol and oxyresveratrol on KA-induced neuronal cell death in the CA3 region in the hippocampus.

## Materials and methods

These experiments were approved by the University of Hallym Animal Care and Use Committee (Registration Number: Hallym 2014–47). All procedures were conducted in accordance with the ‘Guide for Care and Use of Laboratory Animals’ published by the National Institutes of Health and the ethical guidelines of the International Association for the Study of Pain.

### Animals

Male ICR mice (MJ Co., Seoul, South Korea) weighing 20–25 g were used for all the experiments. Animals were housed 5 per cage in a room maintained at 22 ± 0.5°C with an alternating 12 h light–dark cycle. Food and water were available *ad libitum*. The animals were allowed to adapt to the laboratory for at least 2 h before testing and were only used once. Experiments were performed during the light phase of the cycle (10:00–17:00).

### Intracerebroventricular (i.c.v.) injection

The i.c.v. injection was made according to the procedure of Haley and McCormick (1957), using a 30-gauge needle connected by polyethylene tubing to a 25 µl Hamilton. The i.c.v. injection volume was 5 µl, and the injection sites verified by injecting a similar volume of 1% methylene blue solution and determining the distribution of the injected dye in ventricular space. The i.c.v. injected dye was found to be distributed throughout the ventricular spaces, reaching the ventral surface of the brain and upper cervical portion of the spinal cord. The success rate for the injections prior to the experiments was consistently found to be over 95%.

### Cresyl violet staining method and histological analysis

Animals were sacrificed for the brain sample by perfusion at 24 h after phosphate-buffered saline (PBS) or KA administration. All perfusion procedures were worked in the fume hood. For perfusion, all mice were first deeply anesthetized with sodium pentobarbital (100 mg/kg, intraperitoneally, Hanlim Pharm Co, Seoul, Korea) and perfused intracardially with physiological saline followed with ice-cold phosphate-buffered 4% paraformaldehyde (pH 7.4). The whole brain was removed from the skull and post-fixed in the same fixative for 4 h at 4°C. Then the brains were cryoprotected in 30% sucrose for 24 h at 4°C and sectioned coronally (45 μm) on a freezing microtome and collected in cryoprotectant for storage at −20°C until processed. Prepared sections were rinsed 3 × 10 min in PBS to remove cryoprotectant. Sections were mounted on microscope slides (Fisher, Fair Lawn, NJ, USA) and dried on air. The slides were soaked in cresyl violet working solution (0.02% buffer solution; 0.2% sodium acetate, 0.3% acetic acid) for 30 min. Then the sections were dehydrated through alcohol and xylene and coverslipped using Permount (Fisher, USA).

Histological analysis method in pyramidal layer of the hippocampal CA3 region was performed following under procedures. The number of cresyl violet-positive neurons was counted by two blinded observers at the same time using an image analyzing system equipped with a computer-based CCD camera (Olympus AX70; Center Valley, PA, USA). The number of cresyl violet-positive neurons in the CA3 region of the hippocampus was counted in three sections in reference to the mouse atlas for each animal. Starting from the first section (interaural 2.10 mm, bregma – 1.70 mm), counts were taken from at least three coronal sections at 0.135 mm increments. Thus, we could always perform neuronal counting of the same brain region and minimize any counting bias. The number of cresyl violet-positive neurons was compared to that of the control group of the same brain area from all animals. All experiments were conducted independently twice. The neuronal counting of the same group was combined for final analysis.

### Immunohistochemistry

Sections were cut with a cryostat at a thickness of 45 µm. Immuno-histochemical staining was performed with Elite ABC Kit (Vector Laboratories, USA). Sections were first rinsed with 0.1 M PBS three times for 10 min each, then pre-incubated in 0.1 M PBS containing 1% BSA and 0.2% Triton X-100 for 30 min. After rinsing twice with 0.1 M PBS containing 0.5% BSA for 10–15 min each, sections were incubated with antibody against FoxO3a (1:200, AB Frontier, Korea) and pFoxO3a (Phospho-Ser253, 1:200, AB Frontier, Korea) diluted with 0.1 M PBS containing 0.5% BSA and 0.05% sodium azide at 4°C. After overnight incubation, sections were rinsed and incubated with biotinylated secondary antibody 1:200 diluted with 0.1 M PBS containing 0.5% BSA for 1 h at room temperature. After rinsing, the sections were incubated with ABC reagent 1:200 diluted with PBS for 1 h at room temperature and then rinsed with PBS followed with 0.1 M phosphate buffer (PB). Finally, sections were incubated in SIGMA FAST DAB kit (Sigma, USA), until the desired stain intensity developed. We standardized the lengths of DAB reaction time (10 min for all brain sections) to allow for uniform intensity of staining across the experimental groups. Sections were rinsed with 0.1 M PB, and then mounted to gelatin-coated slides, and dehydrated through alcohol and xylene. To quantify, we counted FoxO3a, pFoxO3a in each section. The animal number used for immunostaining was 8 per group.

### Drugs

Resveratrol, Oxyresveratrol and KA were purchased from Sigma Chemical Co. (St. Louis, MO, USA). All drugs were prepared just before use.

### Statistical analysis

Statistical analysis was carried out by *t*-test or one-way analysis of variance with a Bonferroni *post hoc* test using GraphPad Prism Version 4.0 for Windows (GraphPad Software, USA). *P* values less than 0.05 were considered to indicate statistical significance. All values were expressed as the mean ± S.E.M.

## Results

### Effect of resveratrol or oxyresveratrol on KA-induced hippocampal neuronal cell death

**Figure 1.**
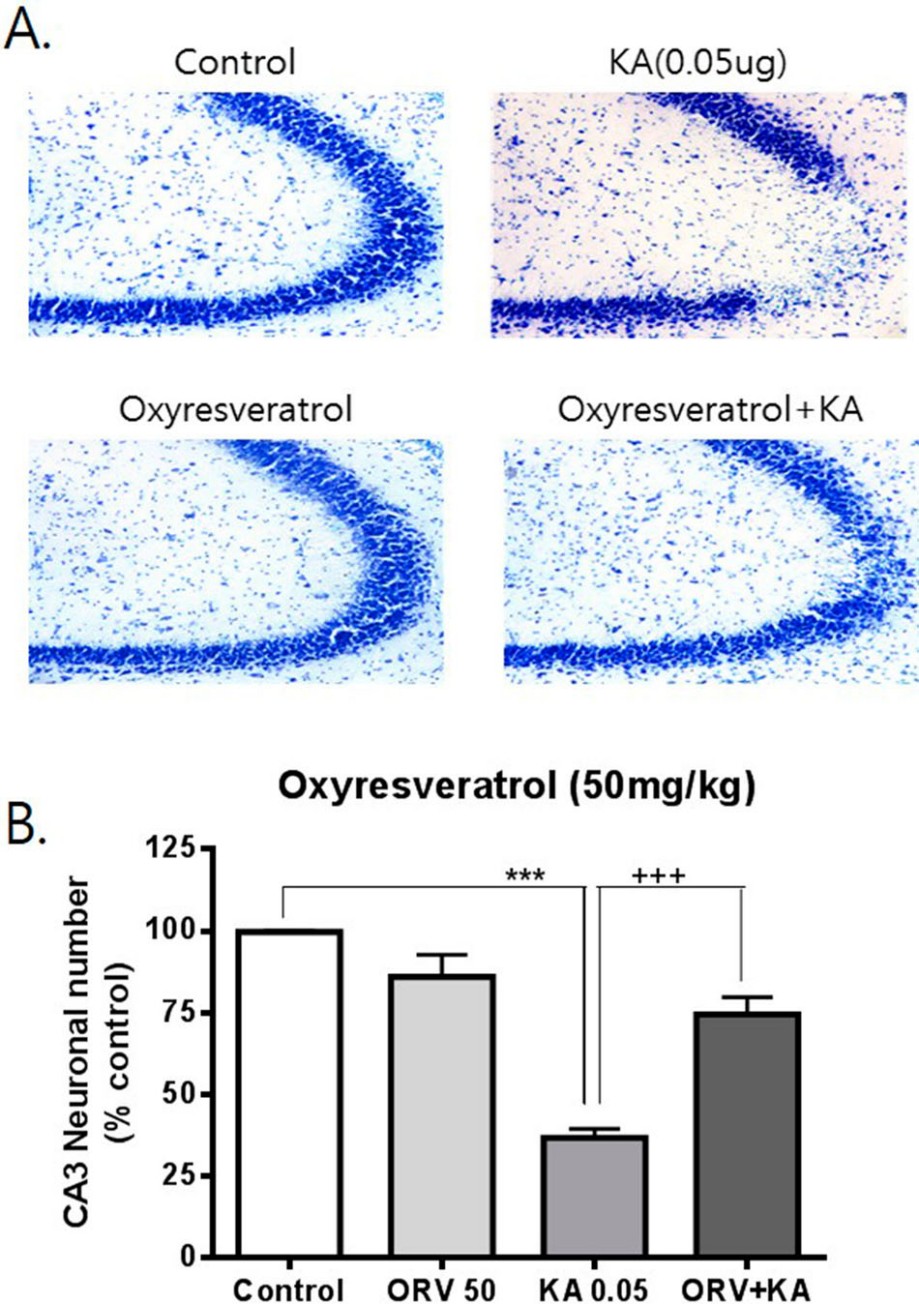
Effect of oxyresveratrol pretreated orally on KA-induced neuronal death in the hippocampus Mice were pretreated orally with 50 mg/kg of oxyresveratrol (ORV) 30 min prior to KA (0.05 μg/5 μl, i.c.v.) administration. The cresyl violet staining was performed at 24 h after KA administration, and then, the cresyl violet-positive neuronal count in the hippocampal CA3 region was performed. The vertical bars in the column graph indicate the S.E.M. (***p < 0.001, control vs KA; +++*p *< 0.0001, KA vs ORV+KA). The number of mice used in each group was 5.

**Figure 2.**
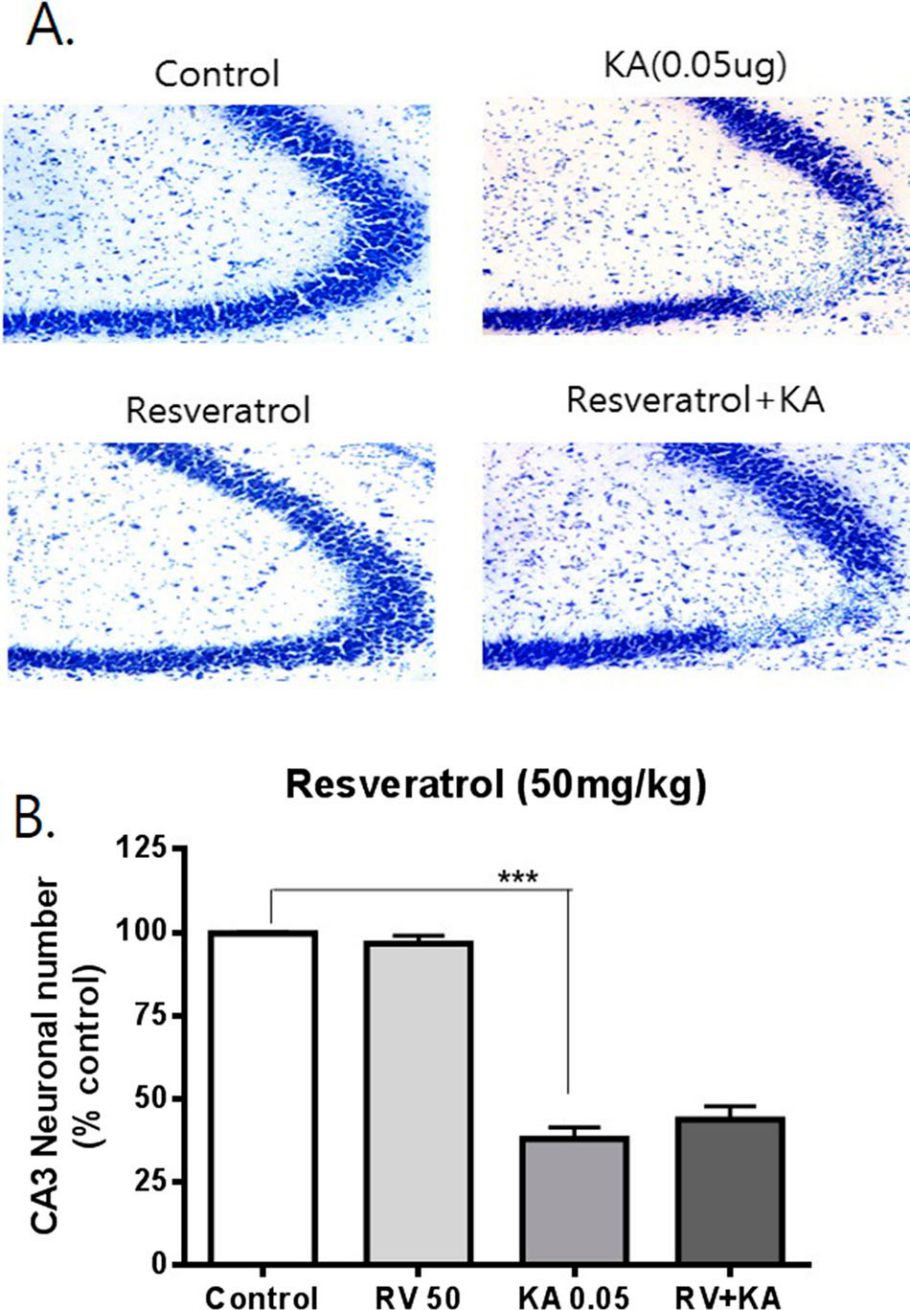
Effect of resveratrol pretreated orally on KA-induced neuronal death in the hippocampus Mice were pretreated orally with 50 mg/kg of resveratrol (RV) 30 min prior to KA (0.05 μg/5 μl, i.c.v.) administration. The cresyl violet staining was performed at 24 h after KA administration, and then, the cresyl violet-positive neuronal count in the hippocampal CA3 region was performed. The vertical bars in the column graph indicate the S.E.M. (***p < 0.001, control vs KA). The number of mice used in each group was 5.

**Figure 3.**
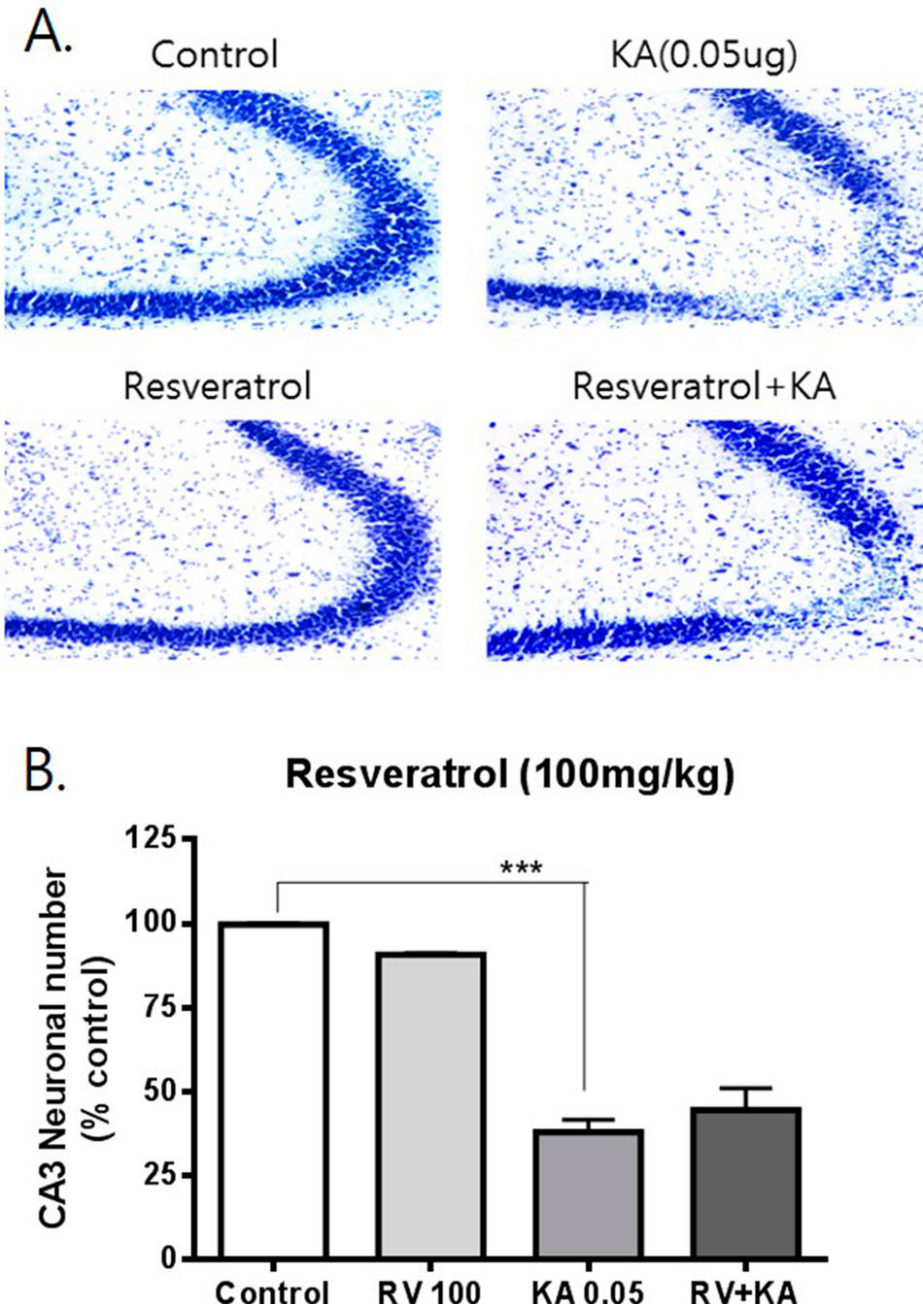
Effect of resveratrol on KA-induced neuronal death in the hippocampus Mice were pretreated orally with 100 mg/kg of resveratrol (RV) 30 min prior to KA (0.05 μg/5 μl, i.c.v.) administration. The cresyl violet staining was performed at 24 h after KA administration, and then, the cresyl violet-positive neuronal count in the hippocampal CA3 region was performed. The vertical bars in the column graph indicate the S.E.M. (***p < 0.001, control vs KA). The number of mice used in each group was 5.

**Figure 4.**
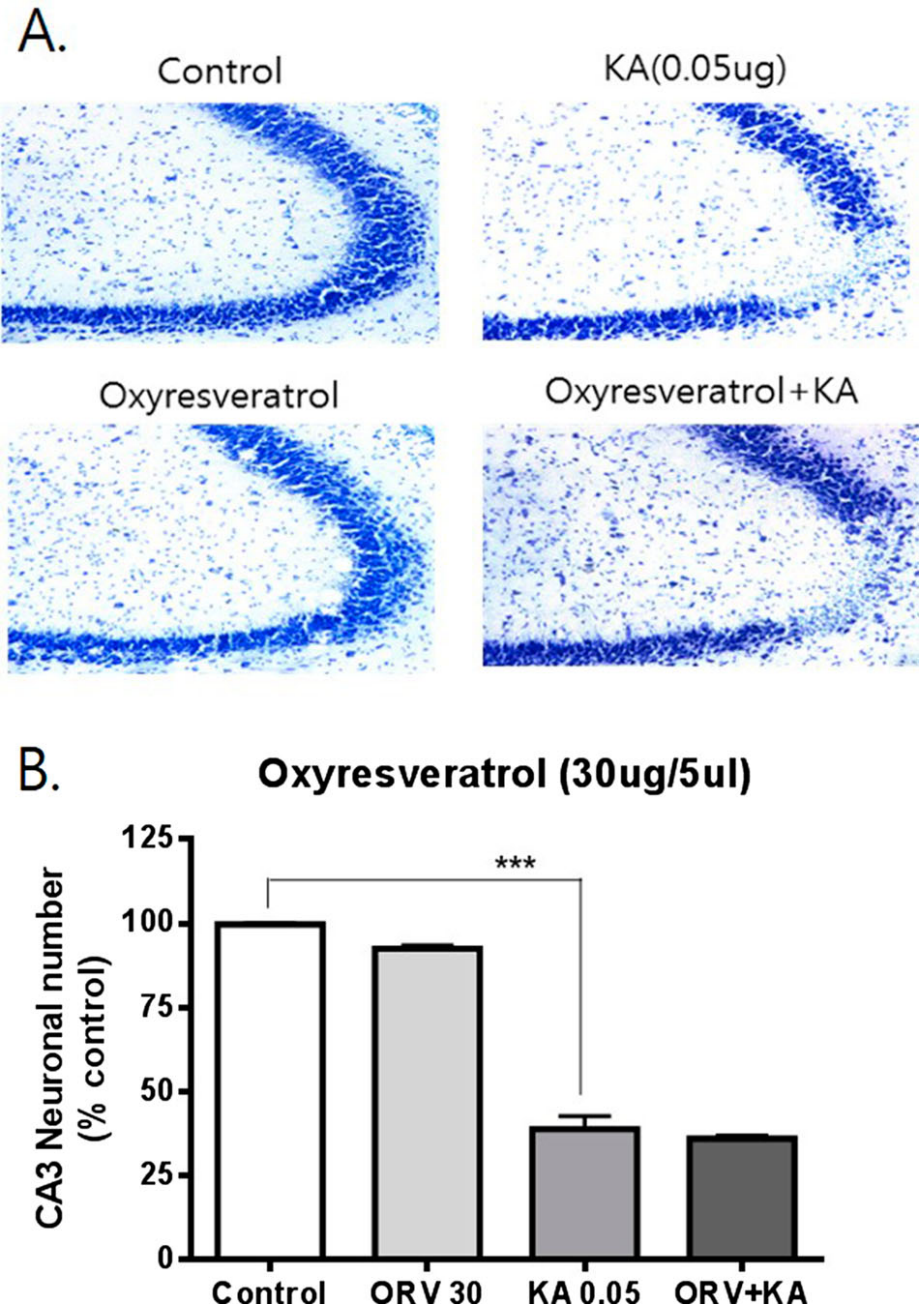
Effect of oxyresveratrol pretreated i.c.v. on KA-induced neuronal death in the hippocampus Mice were pretreated i.c.v. with 30 μg of oxyresveratrol (ORV) 10 min prior to KA (0.05 μg/5 μl, i.c.v.) administration. The cresyl violet staining was performed at 24 h after KA administration, and then, the cresyl violet-positive neuronal count in the hippocampal CA3 region was performed. The vertical bars in the column graph indicate the S.E.M. (***p < 0.001, control vs KA). The number of mice used in each group was 5.

**Figure 5.**
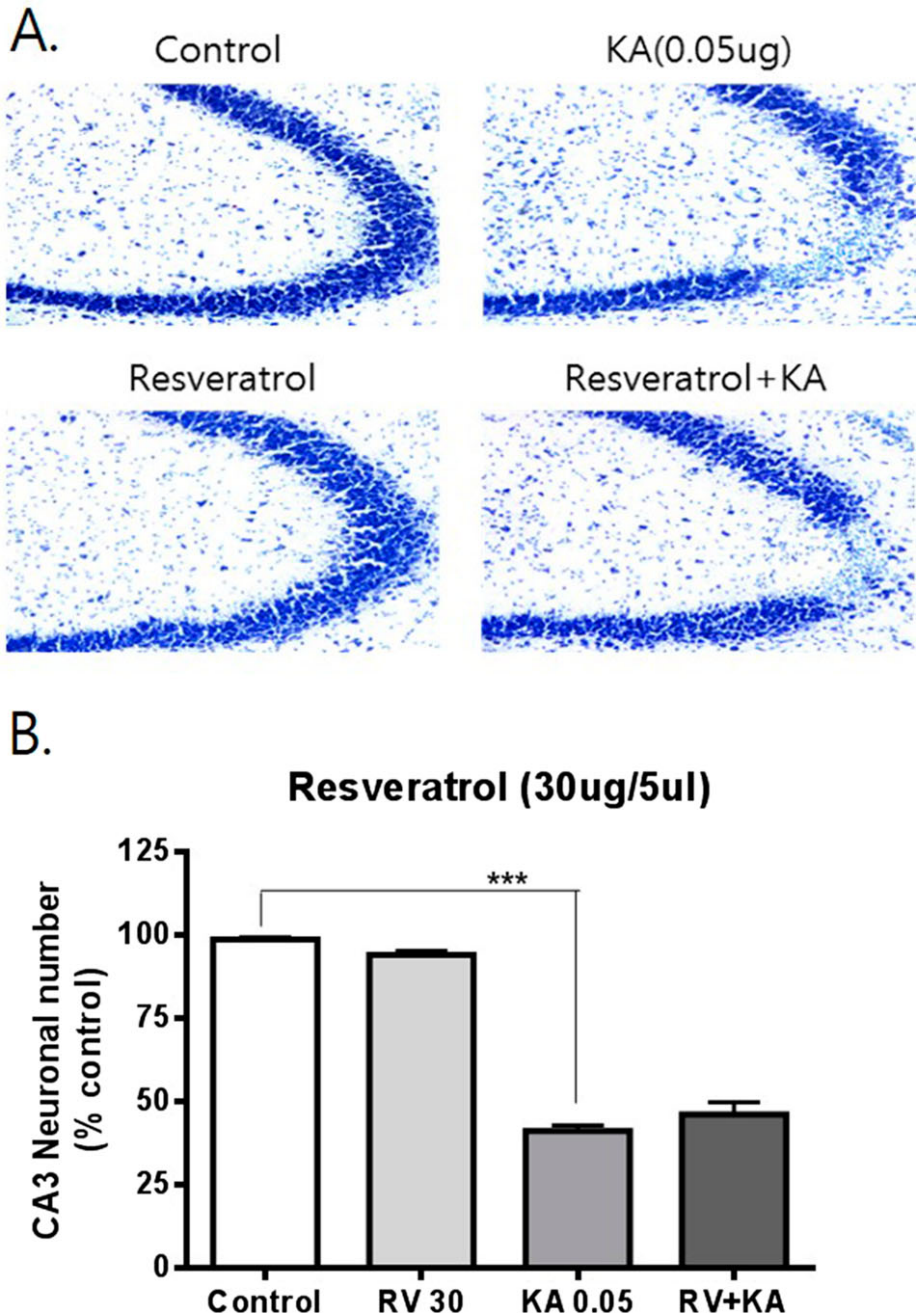
Effect of resveratrol pretreated i.c.v. on KA-induced neuronal death in the hippocampus Mice were pretreated i.c.v. with 30 μg of resveratrol (RV) 10 min prior to KA (0.05 μg/5 μl, i.c.v.) administration. The cresyl violet staining was performed at 24 h after KA administration, and then, the cresyl violet-positive neuronal count in the hippocampal CA3 region was performed. The vertical bars in the column graph indicate the S.E.M. (***p < 0.001, control vs KA). The number of mice used in each group was 5.

To examine the possible effects of resveratrol and oxyresveratrol against KA-induced hippocampal neuronal cell death, mice were administered orally with resveratrol or oxyresveratrol 30 min prior to i.c.v. administration with KA (0.05 μg). As shown in Figure 1, oral pretreatment with oxyresveratrol at a dose of 50 mg/kg significantly attenuated KA-induced neuronal cell death in the CA3 region of the hippocampus. However, the same dose (50 mg/kg) or even a higher dose (100 mg/kg) pretreatment with resveratrol did not affect KA-induced hippocampal neuronal cell death (Figures 2 and 3). To examine if oxyresveratrol exerts the neuroprotective effect by direct action on the central nervous system, mice were pretreated i.c.v. with either resveratrol or oxyresveratrol for 10 min prior to i.c.v. administration with KA. As shown in Figures 4 and 5, the i.c.v. pretreatment with 30 μg of oxyresveratrol or resveratrol did not alter the KA-induced hippocampal neuronal cell death in CA3 region.

### Effect of oxyresveratrol on KA-induced FoxO3a and pFoxO3a expressions in CA3 region of the hippocampus

**Figure 6.**
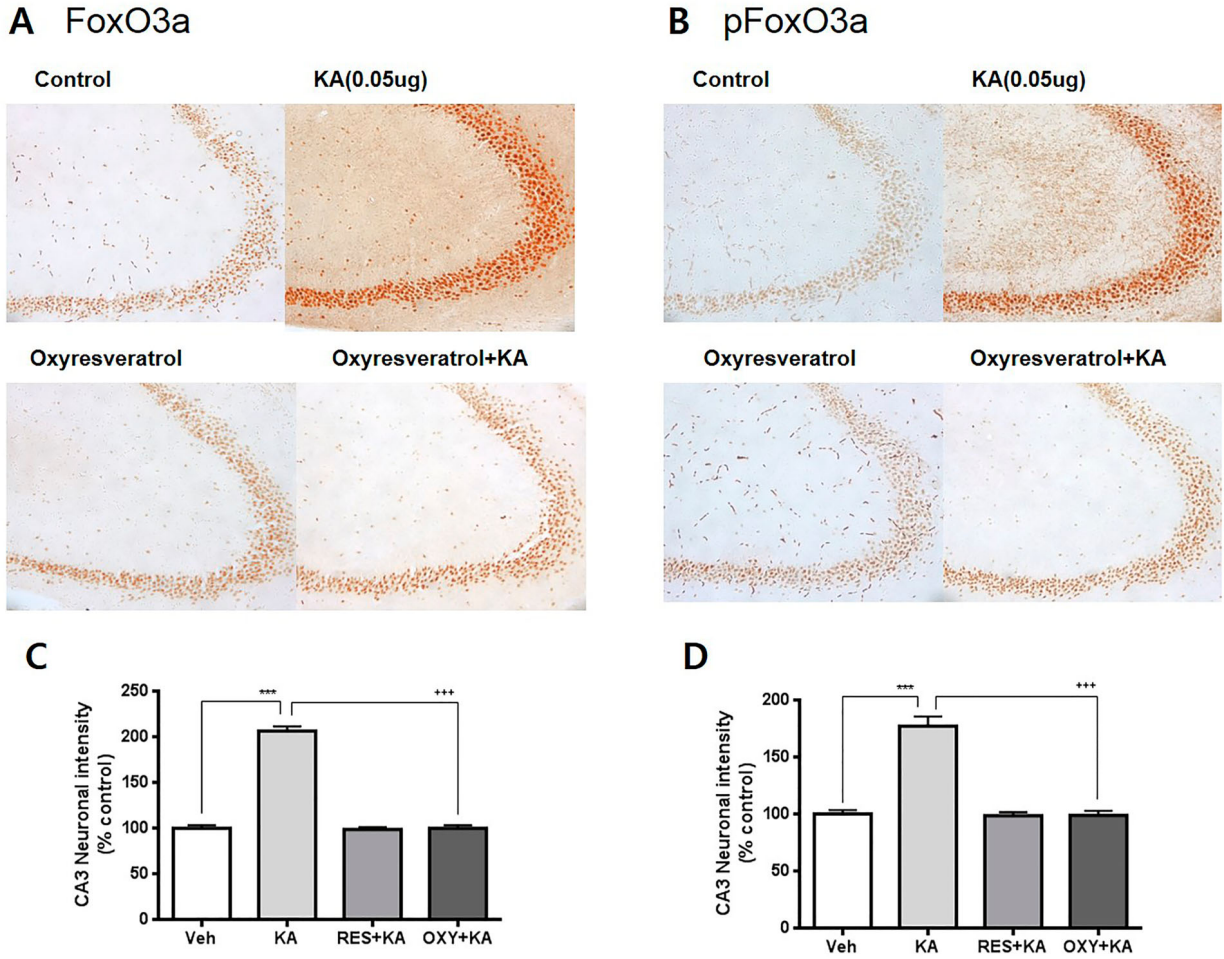
Effect of oxyresveratrol pretreated orally on KA-induced FoxO3a and pFoxO3a expressions in the hippocampal CA3 region. An immunohistochemical study for FoxO3a and pFoxO3a in the hippocampal CA3 region (A, C) was carried out. Oxyresveratrol (ORV) was pretreated orally (50 mg/kg) 30 min prior to KA (0.1 μg/5 μl, i.c.v.) administration. Immunohistochemistry study was performed 0.5 h after KA administration. The FoxO3a and pFoxO3a positive cells in the hippocampaal CA3 region were counted referencing to the mouse atlas. The vertical bars in the column graph indicate the standard error of the means (B, D). (***p < 0.001, control vs KA; +++*p *< 0.001, KA vs ORV+KA). The number of mice used in each group was 5.

To examine the possible role of FoxO3a protein in neuroprotective effect of oxyresveratrol against KA-induced neurotoxicity, the effect of oxyresveratrol on KA-induced FoxO3a and phosphorylated FoxO3a (pFoxO3a) in the hippocampal CA3 region was examined. The FoxO3a and pFoxO3a positive cells in the hippocampal CA3 region were counted referencing to the mouse atlas at 0.5 h after KA administration. As shown in Figure 6, KA caused increases of the expression of FoxO3a and pFoxO3a in the hippocampal CA3 region. Oxyresveratrol (50 mg/kg) was orally pretreated 30 min prior to KA administration. Then, the expressions of FoxO3a and pFoxO3a in the hippocampal CA3 region were observed at 0.5 h after KA administration. Oxyresveratrol treatment alone did not alter FoxO3a and pFoxO3a in the hippocampal CA3 region. Oral pretreatment with oxyresveratrol attenuated KA-induced FoxO3a and pFoxO3a expressions in the hippocampal CA3 region (Figure 6).

## Discussion

The results of the present study showed that oral pretreatment with oxyresveratrol exerts a neuroprotective effect against KA-induced hippocampal CA3 neuronal cell death. However, when the same dose (50** **mg/kg) or even at higher dose (100** **mg/kg) of resveratrol was pretreated, resveratrol did not affect KA-induced neurotoxicity. Although several lines evidence have demonstrated that resveratrol shows some neuroprotective effects (Wang et al. 2004; Liu et al. 2016), the result of the present study suggests that orally treated resveratrol is not effective in for KA-induced neurotoxicity. However, our result is in part line with a previous study in that oxyresveratrol shows a neuroprotective action and inhibits the apoptotic cell death in transient rat middle cerebral artery occlusion model of brain ischemia (Andrabi et al. 2004).

In contrast to the results with orally administered oxyresveratrol, no neuroprotective effect was shown when oxyresveratrol was pretreated supraspinally. This result suggests that oxyresveratrol exerts its neuroprotective effect after conversion into some metabolites rather than direct effect on the central nervous system. Although a study shows oxyresveratrol may cross the blood–brain barrier in healthy rats and shows neuronal protective effect in subjects submitted to focal infarction (Breuer et al. 2006), our result clearly suggests that oxyresveratrol may be metabolized into other forms of molecules peripherally or centrally. Huang et al. (2010) have identified some metabolites of oxyresveratrol after oral administration in rat. The exact metabolite responsible for oxyresveratrol-induced neuroprotection against KA-induced neurotoxicity should be clarified in the future study.

Although resveratrol was not effective to exert an inhibitory effect against KA-induced neuronal toxicity as revealed in our study, some previous studies such as Gupta et al. (2002) and Mishra et al. (2015) reported the neuroprotective effects of resveratrol after status epilepticus (SE). Both in their experiments, multiple doses of resveratrol were treated after SE in rats. Resveratrol was injected intraperitoneally in 5 min prior and repeated 30 and 90 min after SE in Gupta et al. group (2002), whereas rats received hourly injections of resveratrol for three hours in Mishra et al. group (2015), which commenced an hour after the onset of SE. According to these pieces of evidence, we inferred that these differential effects of resveratrol may result from the differential origins of animal, species, or the dose and route of drug administration.

FoxO3a may play a significant role during injuries that involve cerebral ischemia and oxidative stress (Cheng and Sun 1994; Chong et al. 2005). FoxO3a transcription is induced by cellular hypoxia via direct binding of the hypoxia-inducible factor HIF1 to the FoxO3 promoter (Bakker et al. 2007). Some previous studies have reported that expression of a constitutively nuclear FoxO3a can promote the death of purified motor neurons and cerebellar granule cells (Brunet et al. 1999; Barthélémy et al. 2004). In a recent study, we have reported that FoxO3a expression is increased in the CA3 region in the hippocampus by i.c.v. injection with KA and is attenuated by the pretreatment with either *N*-methyl-d-aspartate (NMDA) or non-NMDA receptor antagonist, suggesting that FoxO3a protein my play an important role in KA-induced neuronal cell death in the hippocampal CA3 region (Park et al. 2016). However, the role of FoxO3a in the regulation of neuroprotective effect of oxyresveratrol has not been demonstrated in KA-induced hippocampal cell death. Thus, in the present study, oxyresveratrol was pretreated orally 30 min prior to KA administration and we have found that oxyresveratrol pretreated orally attenuated KA-induced expressions of FoxO3a and pFoxO3a in CA3 region of the hippocampus, suggesting that orally administered oxyresveratrol exerts its neuroprotective effect against KA-induced neuronal cell death by reducing the elevated expressions of FoxO3a and pFoxO3a in CA3 region of the hippocampus. A recent report suggested that Akt-FoxO3a signaling plays an important role in the production of neuronal cell death in status epilepticus model (Kim et al. 2014). The same group also has reported that systemic injection with KA increases FoxO3a level whereas KA decreases pFoxO3a expression in CA3 region in the hippocampus. Although the exact reasons for this finding is currently unclear, this differential effect might be due to the differential origins of animal, species, age, route of drug administration, cellular localization, animal model difference, specific antibody kind used in the experiment.
